# Reply: Cetuximab in small bowel adenocarcinoma: a new friend?

**DOI:** 10.1038/sj.bjc.6605899

**Published:** 2010-09-14

**Authors:** M J Overman, R A Wolff, H Wang

**Affiliations:** 1 Department of Gastrointestinal Medical Oncology, The University of Texas MD Anderson Cancer Center, 1515 Holcombe Boulevard, Houston 77030, TX, USA; 2 Department of Pathology, The University of Texas MD Anderson Cancer Center, 1515 Holcombe Boulevard, Houston 77030, TX, USA

**Sir,**

We appreciate the comments and data provided by Santini *et al* based upon our recent report (Overman *et al*, 2010; Figure 1). The use of anti-EGFR therapy in small bowel adenocarcinoma (SBA) is rationale, based upon the high-level expression of the target and known activity of this agent in adenocarcinomas of the large intestine. Though small and large intestinal adenocarcinomas differ dramatically in incidence, a number of similarities in clinical behaviour, such as metastatic site predilection and chemotherapy responsiveness, do exist. As activating mutations in the Kras oncogene are critical in determining the activity of anti-EGFR therapy in colorectal cancer, molecular testing for mutations in the *Kras* gene must also be incorporated into the assessment of anti-EGFR therapy in SBA.

In the commentary by Santini *et al*, an impressive radiographic response of 75% was observed in four patients treated with the combination of cetuximab and irinotecan in a primarily Kras wild-type SBA population. As mentioned by the authors, this finding is encouraging and appears improved over the response rates observed with 5-fluorouracil and irinotecan combinations (Zaanan *et al*, 2010). However, the contributory effect of cetuximab cannot be determined as cetuximab was combined with a known active agent in SBA. We have recently treated a 67-year-old man with metastatic moderately differentiated adenocarcinoma of the duodenum to liver and retroperitoneal lymph nodes with single-agent cetuximab (500 mg m^−2^ every other week) as the fourth-line therapy. After 8 weeks, a 24% reduction in tumour size per RECIST criteria was observed. The pre-treatment (A) and post-treatment (B) computed tomography images are shown in accompanying figure. The subsequent treatment course was complicated by cholangitis and radiographic progression occurred after 20 weeks.

We agree with Santini *et al* that further prospective studies are needed to determine the role of anti-EGFR therapy in SBA. In an attempt to build upon our previous work with the combination of capecitabine and oxaliplatin, CAPOX (Overman *et al*, 2009), we are currently initiating a phase II study evaluating the combination of panitumumab with CAPOX as the first-line treatment for advanced SBA with wild-type Kras.

## Figures and Tables

**Figure 1:**
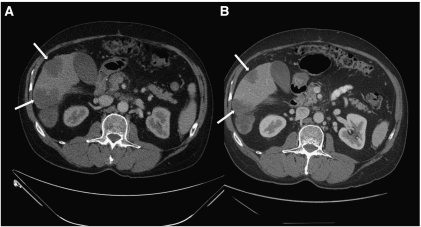
Pre-treatment (**A**) and post-treatment (**B**) computed tomography images showing radiographic response to single agent cetuximab in a patient with metastatic duodenal adenocarcinoma.
